# Supplementary figures and images for: p53 isoforms have a high aggregation propensity, interact with chaperones and lack binding to p53 interaction partners
Source: eLife. 2025 Sep 10;13:RP103537. doi: 10.7554/eLife.103537 (PMC12422735; doi:10.7554/eLife.103537)

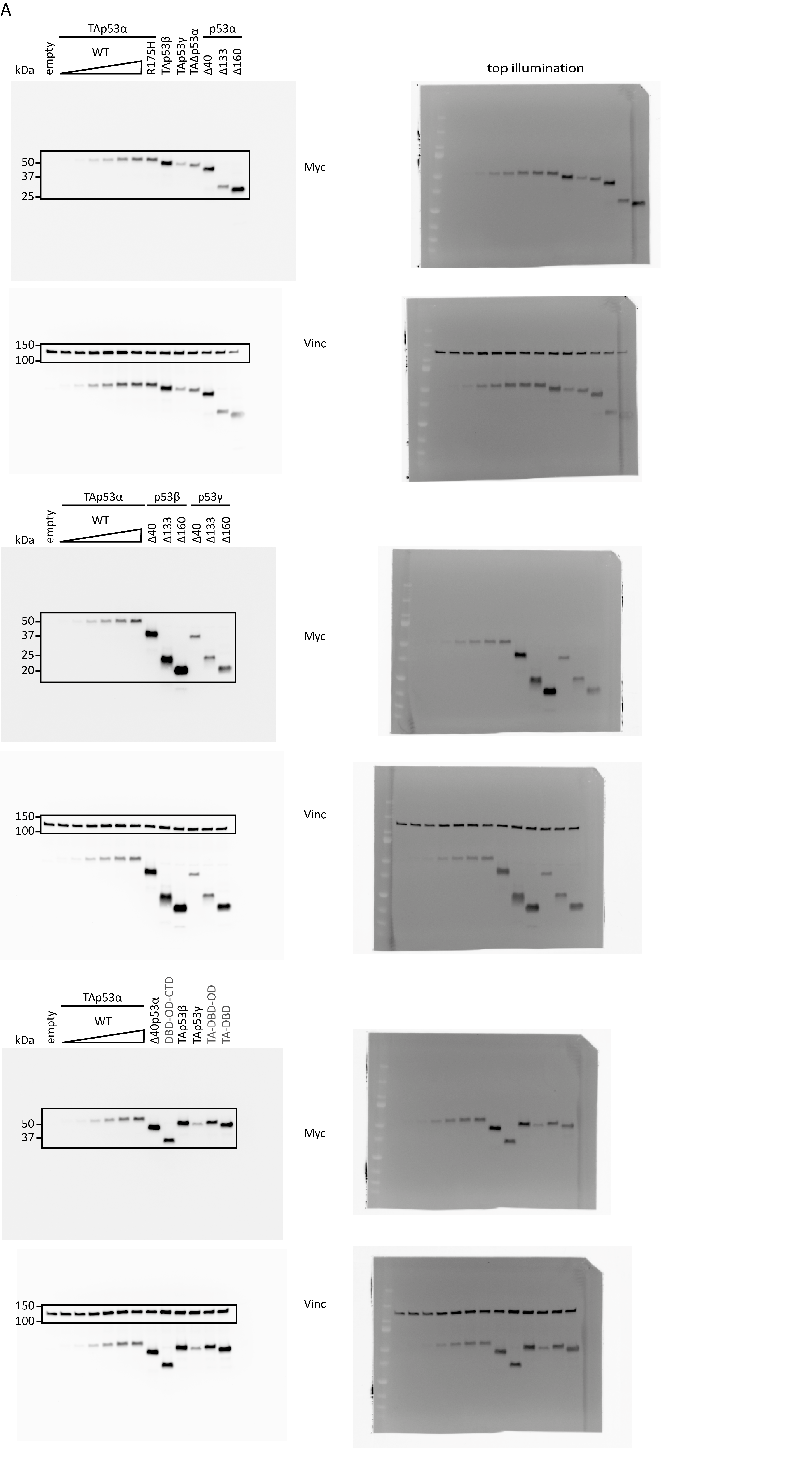

Supplement: Figure 2—figure supplement 1—source data 1. [file elife-103537-fig2-figsupp1-data1.zip › figure 2 - figure supplement 1 - source data A.png]

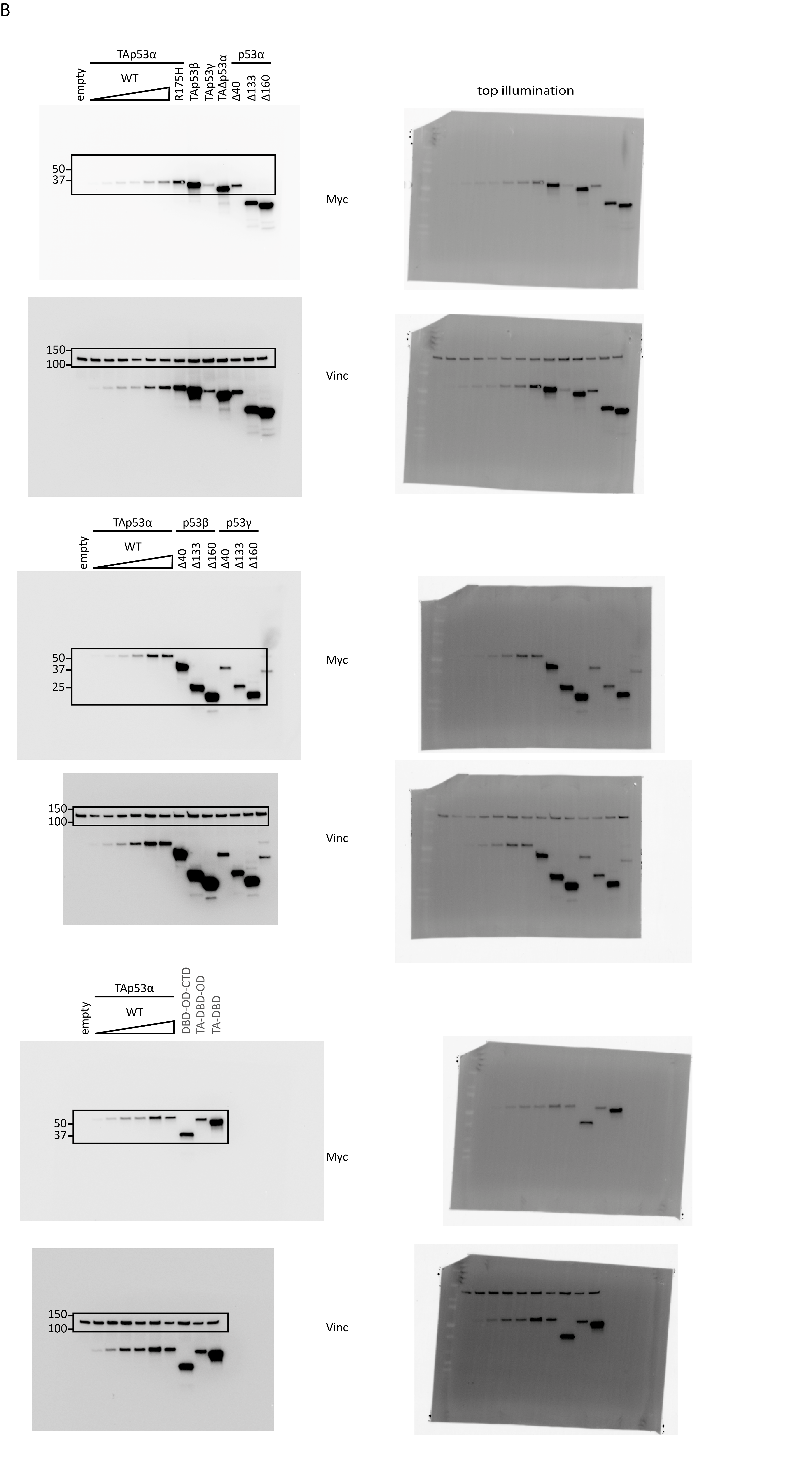

Supplement: Figure 2—figure supplement 1—source data 1. [file elife-103537-fig2-figsupp1-data1.zip › figure supplement 1 - source data B.png]

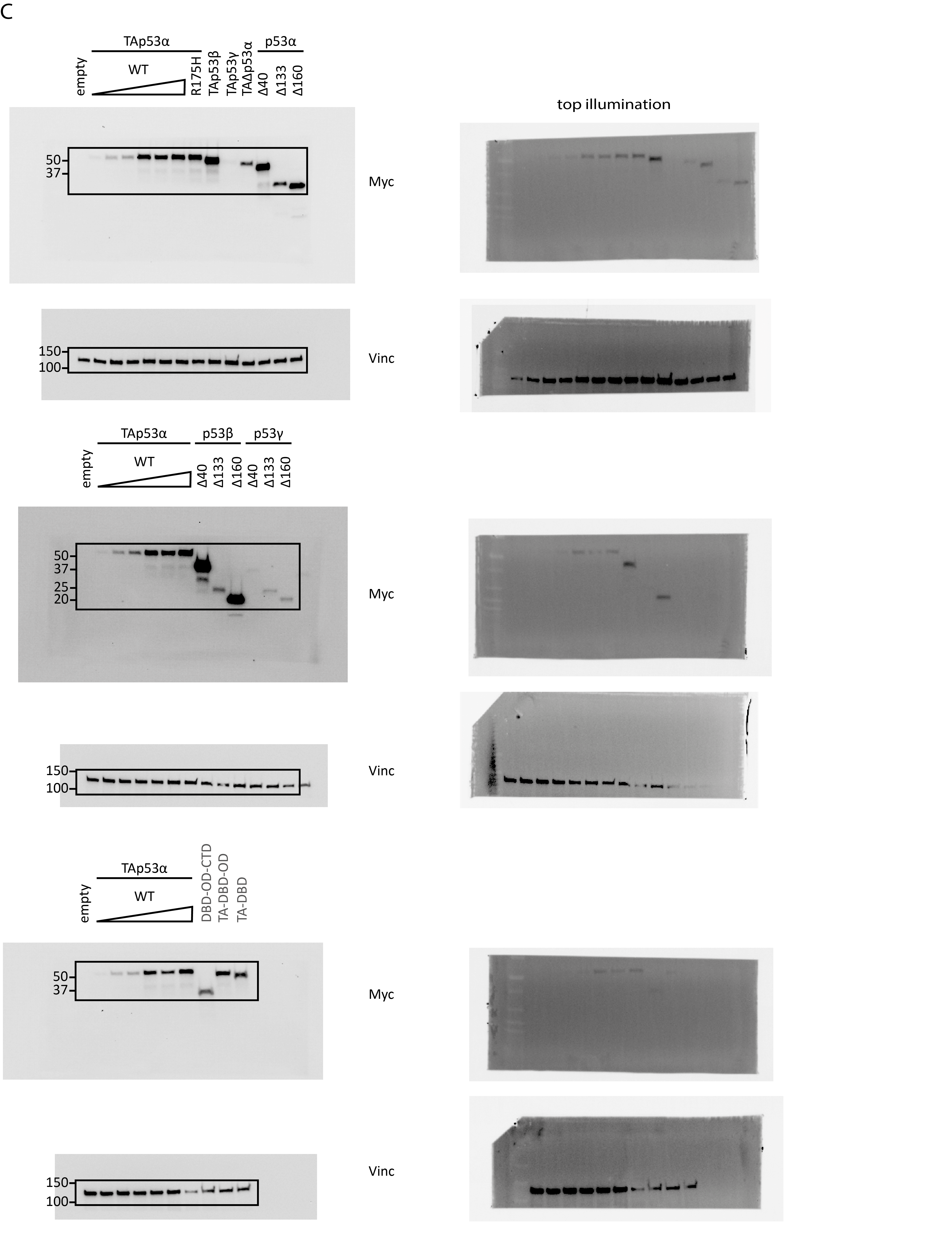

Supplement: Figure 2—figure supplement 1—source data 1. [file elife-103537-fig2-figsupp1-data1.zip › figure supplement 1 - source data C.png]

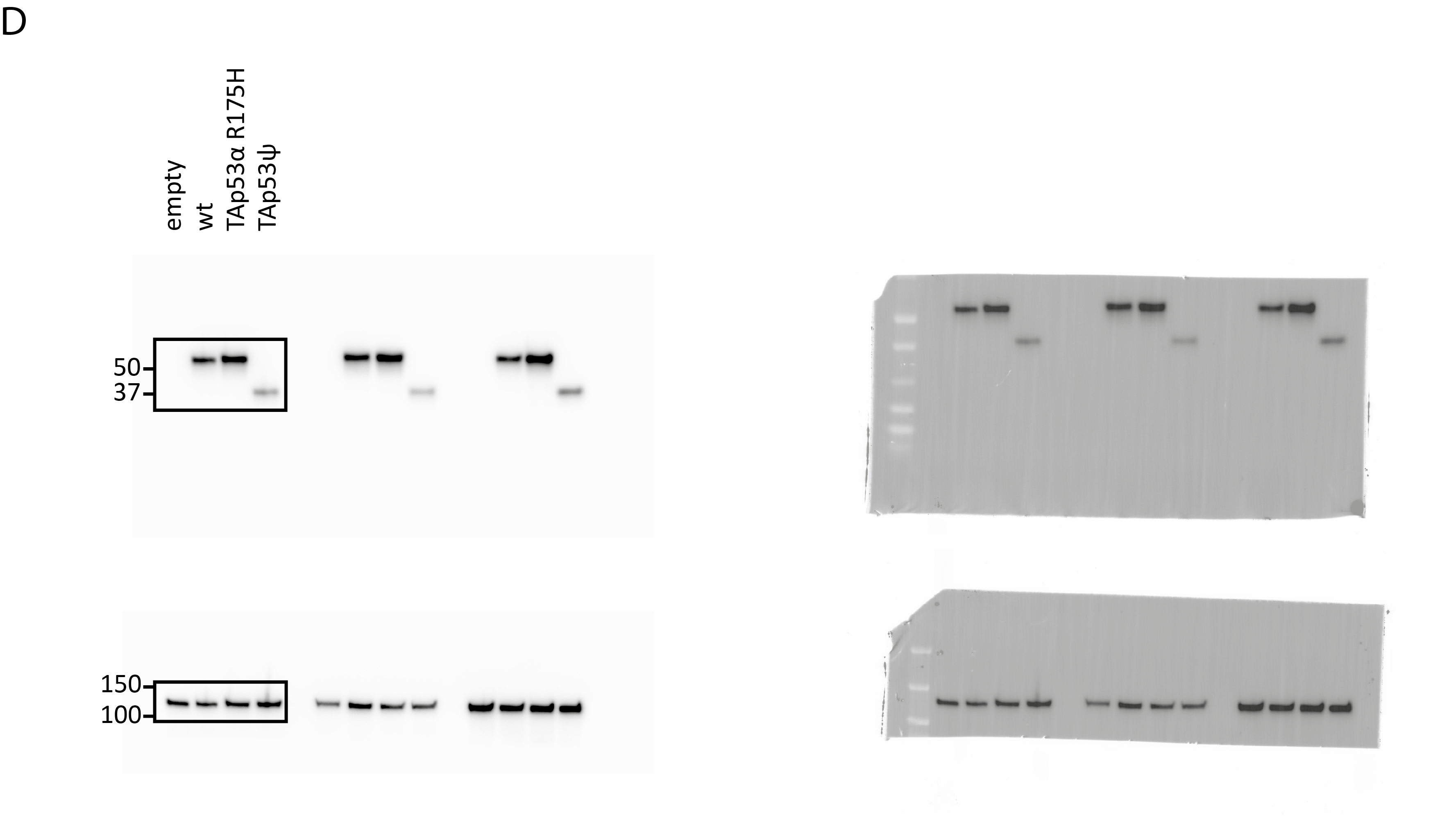

Supplement: Figure 2—figure supplement 1—source data 1. [file elife-103537-fig2-figsupp1-data1.zip › figure supplement 1 - source data D.png]

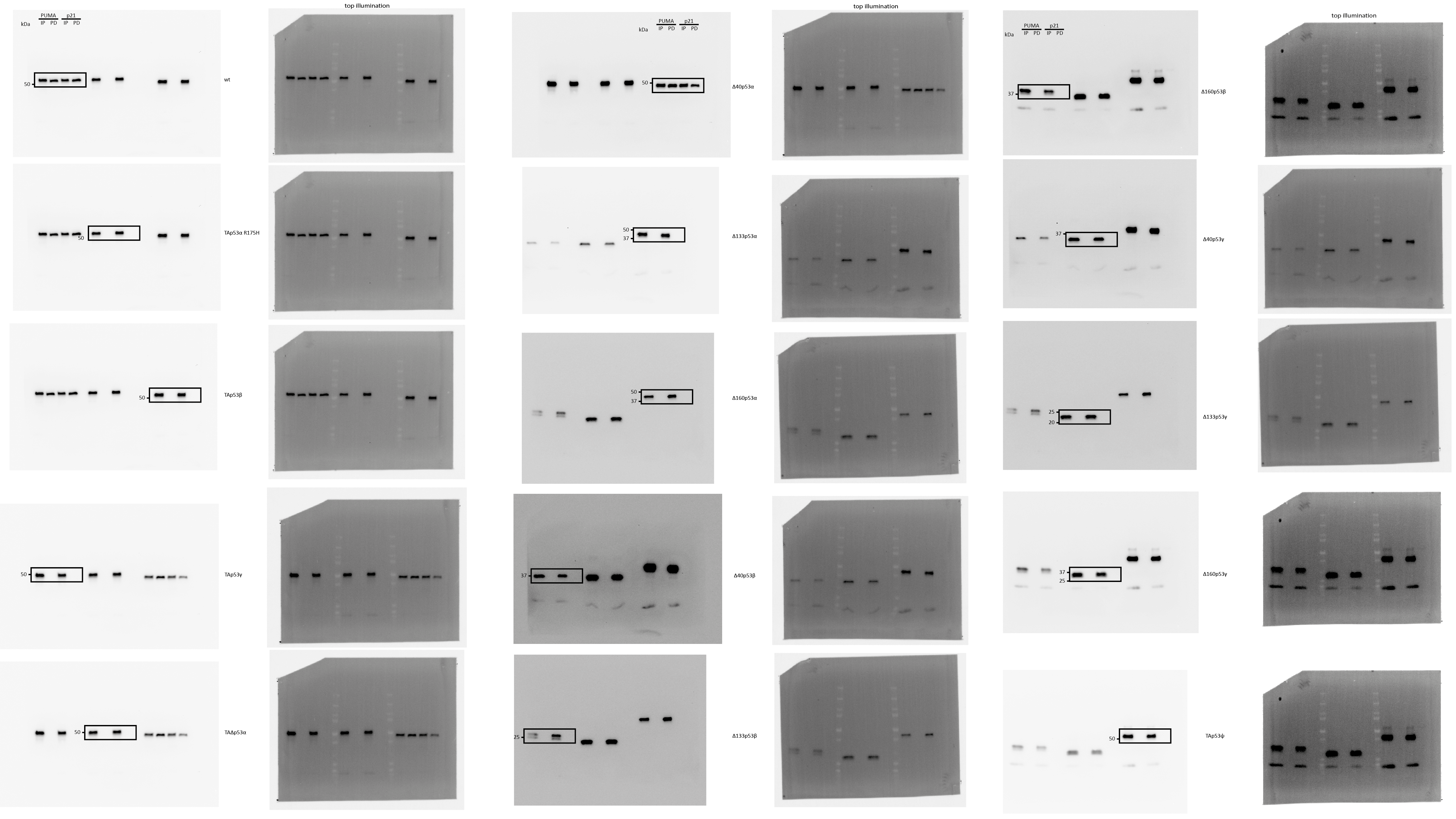

Supplement: Figure 2—figure supplement 2—source data 1. [file elife-103537-fig2-figsupp2-data1.zip › Figure 2 - supplement 2 - source data E.png]

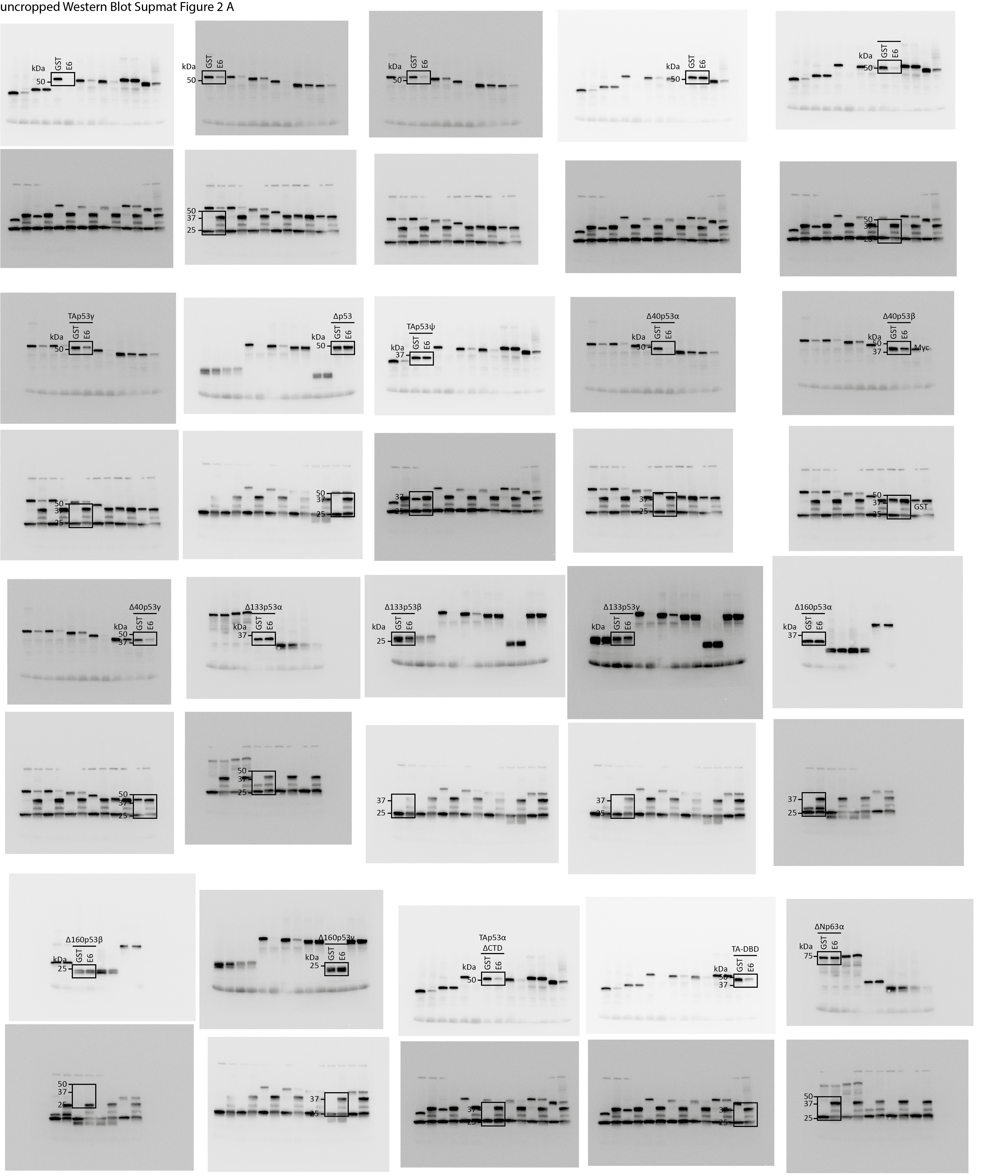

Supplement: Figure 3—figure supplement 1—source data 1. [file elife-103537-fig3-figsupp1-data1.zip › Figure 3 - supplement 1 - source data-01.png]

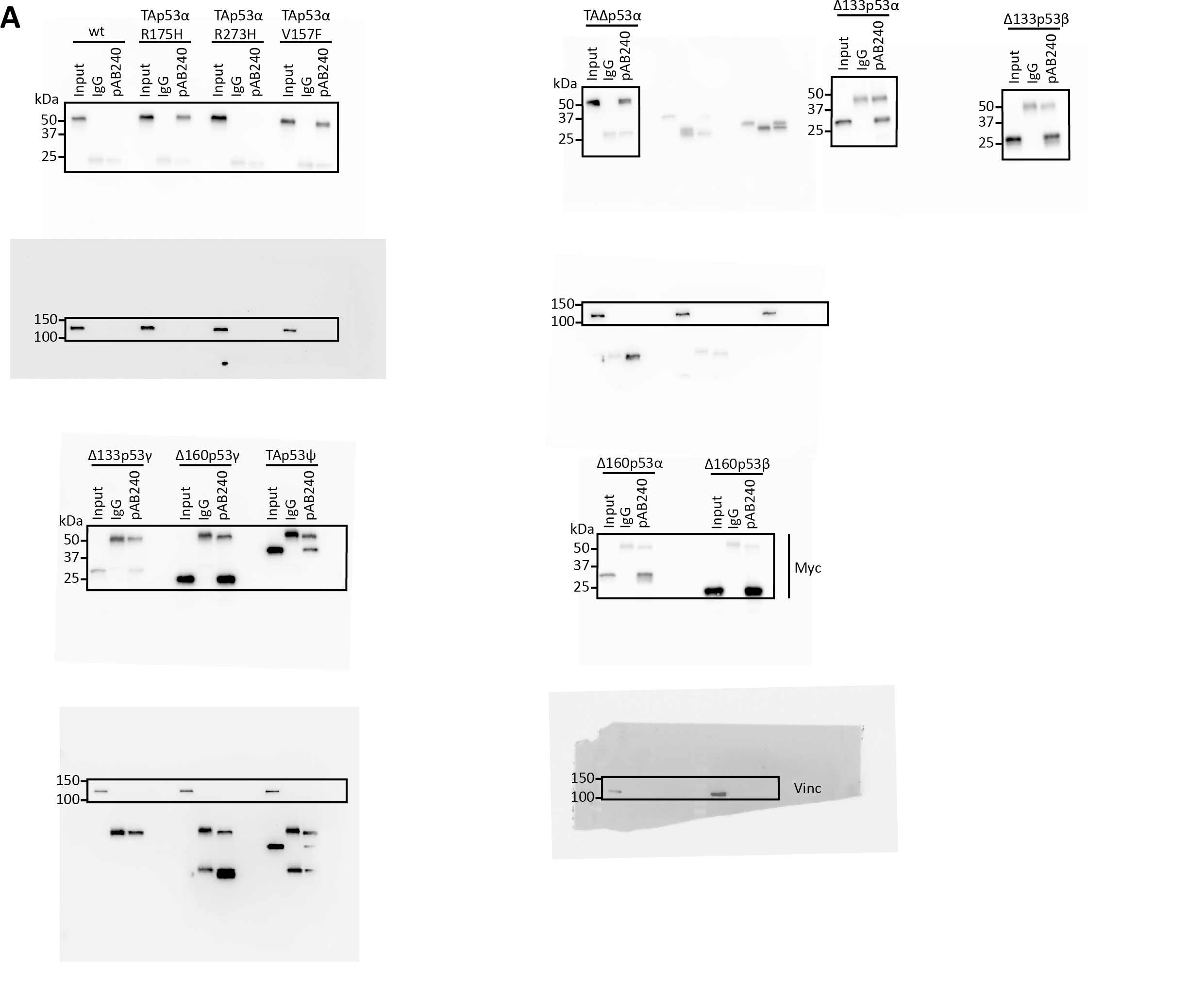

Supplement: Figure 3—figure supplement 2—source data 1. [file elife-103537-fig3-figsupp2-data1.zip › Figure 3 - supplement 2 - source data_A.png]

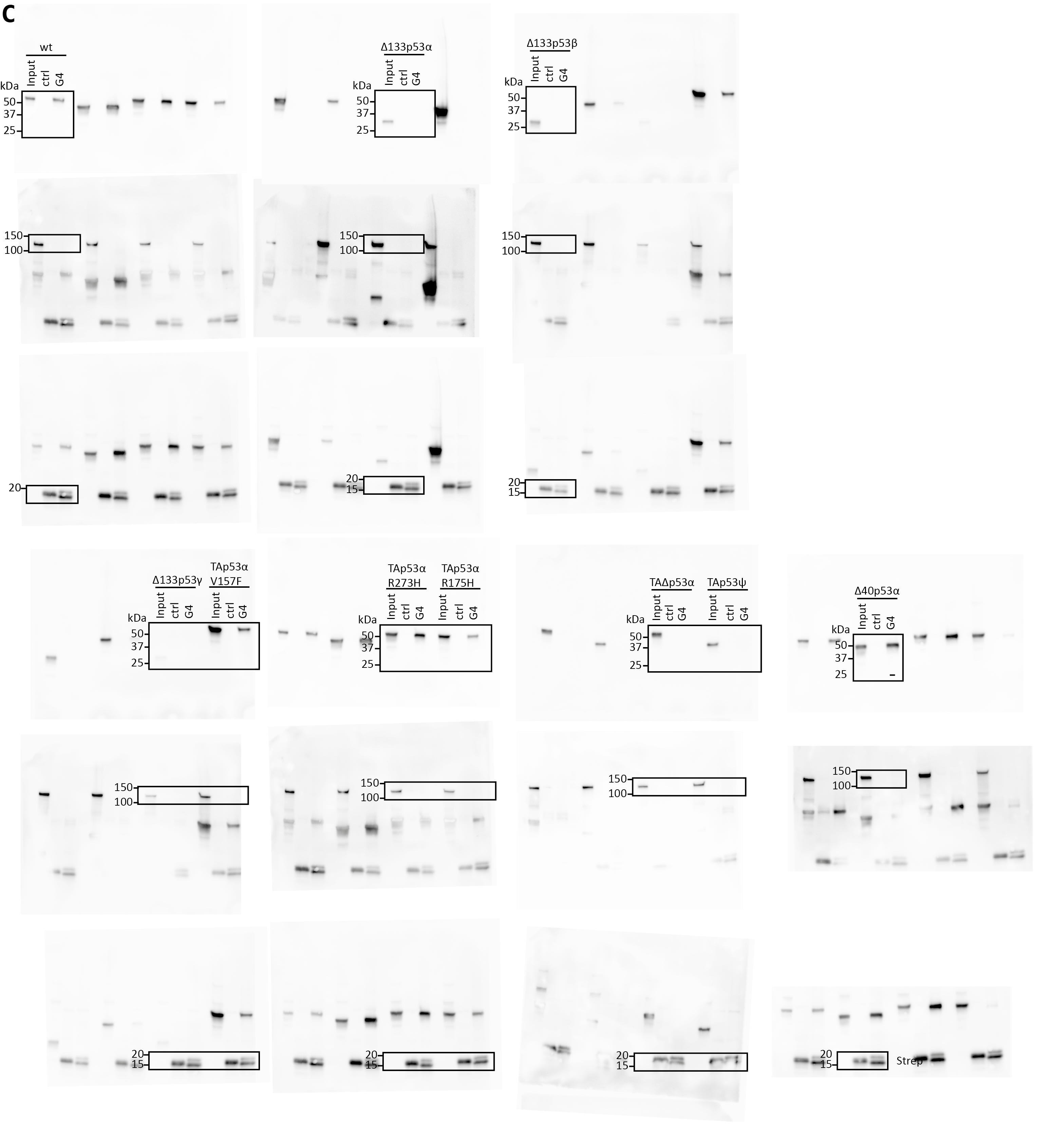

Supplement: Figure 3—figure supplement 2—source data 1. [file elife-103537-fig3-figsupp2-data1.zip › Figure 3 - supplement 2 - source data_C.png]

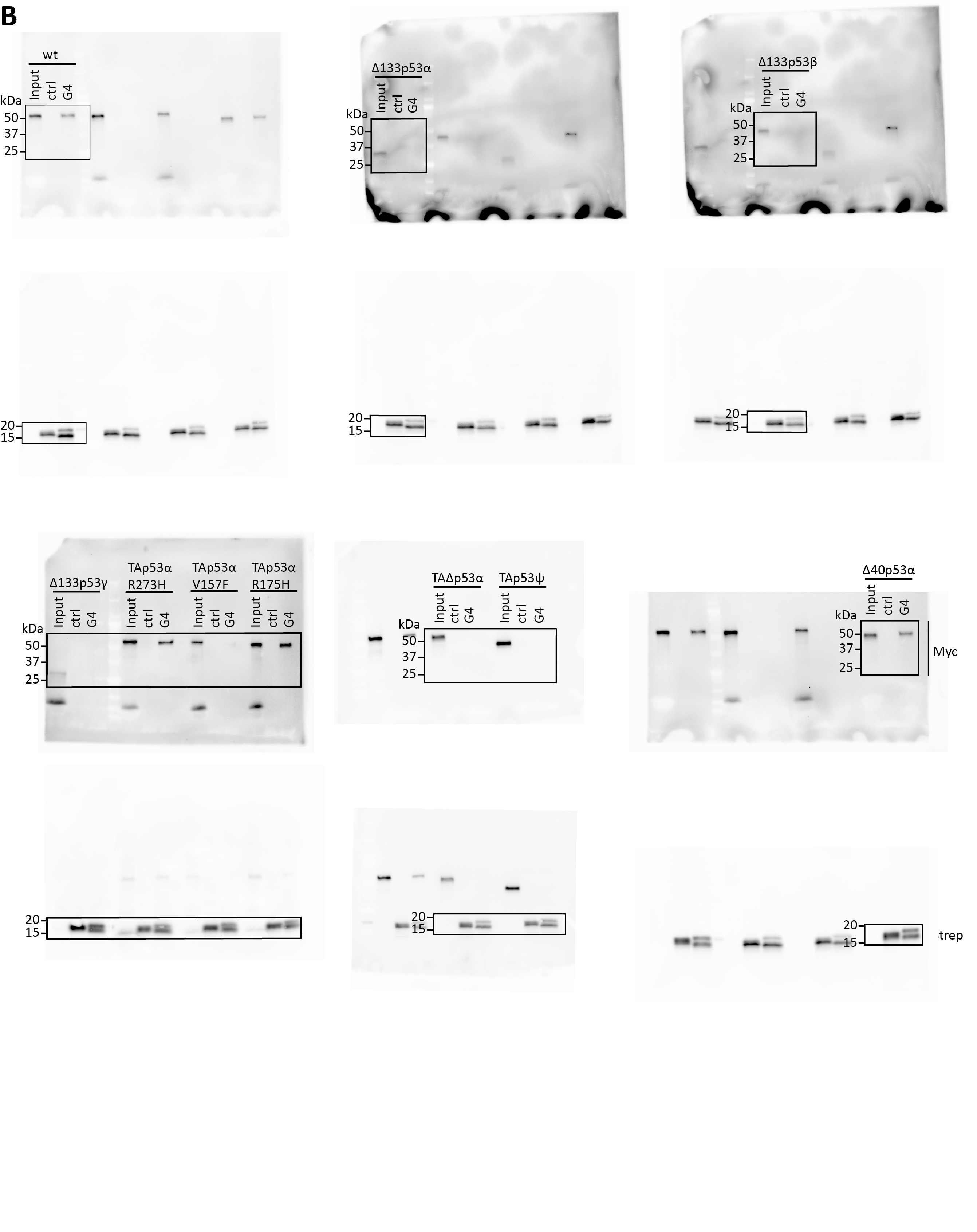

Supplement: Figure 3—figure supplement 2—source data 1. [file elife-103537-fig3-figsupp2-data1.zip › Figure 3 - supplement 2 - source data-B.png]

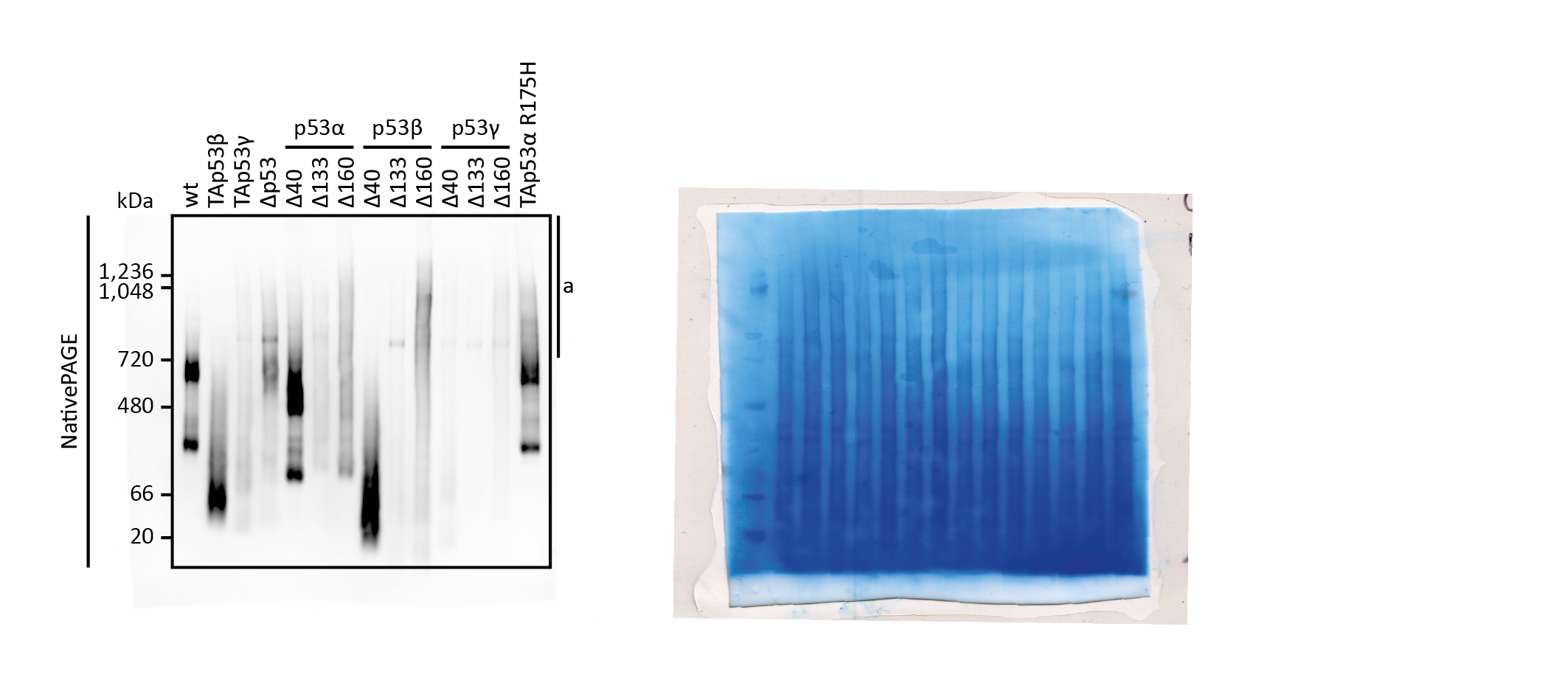

Supplement: Figure 4—source data 1. [file elife-103537-fig4-data1.zip › Figure 4 - source data_E.png]

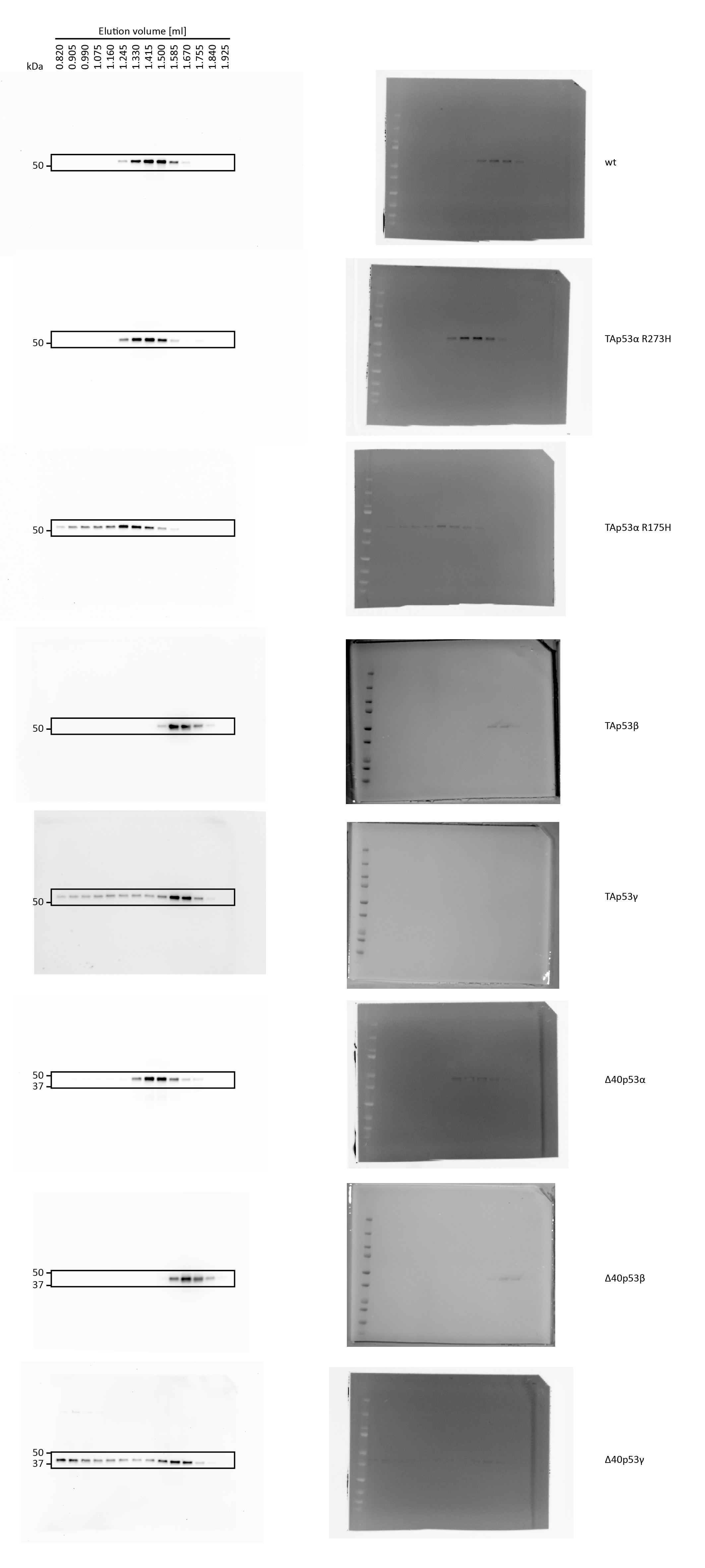

Supplement: Figure 4—source data 1. [file elife-103537-fig4-data1.zip › Figure 4 - source data_D-1.png]

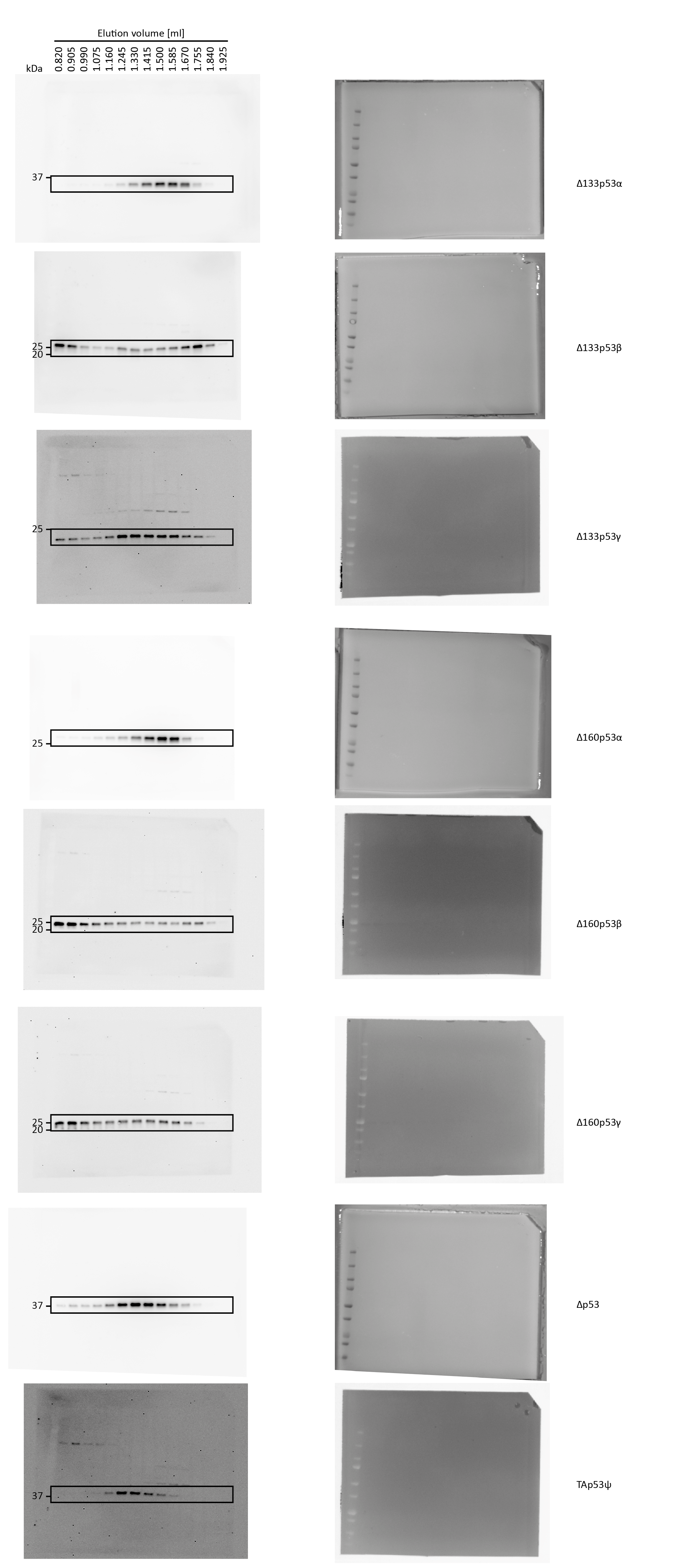

Supplement: Figure 4—source data 1. [file elife-103537-fig4-data1.zip › Figure 4 - source data_D-2.png]

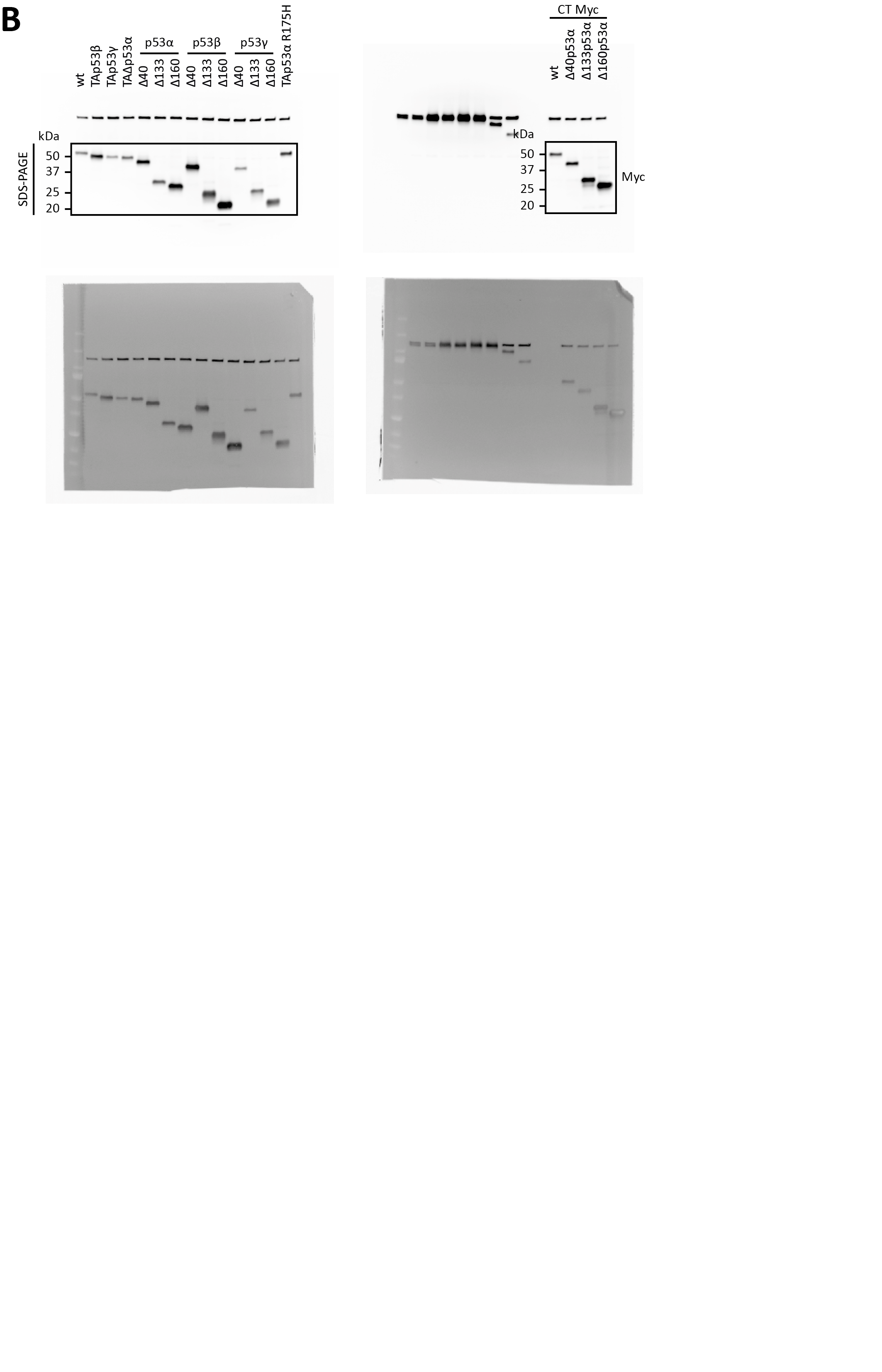

Supplement: Figure 4—figure supplement 1—source data 1. [file elife-103537-fig4-figsupp1-data1.zip › Figure 4 - supplement 1 - source data-02.png]

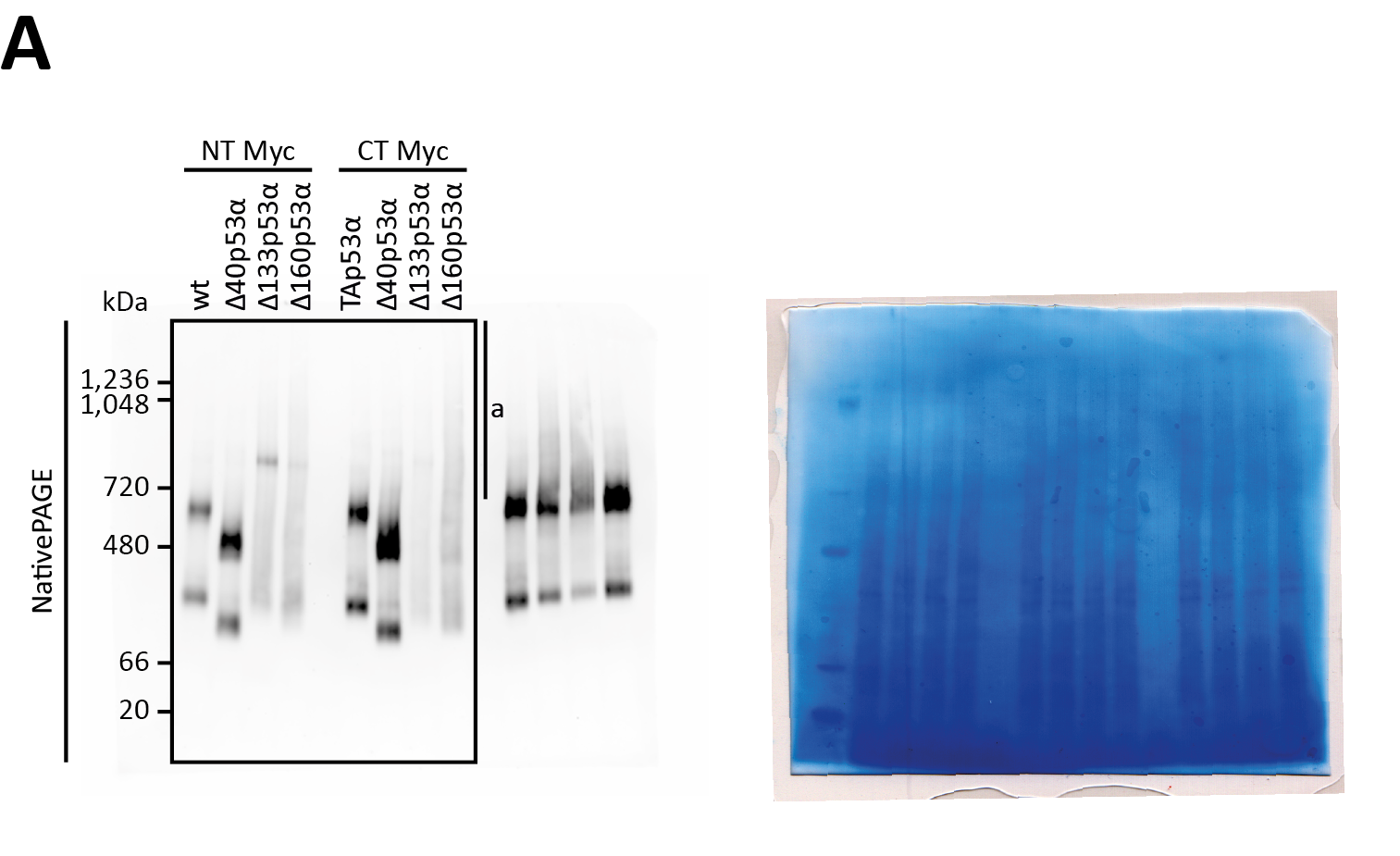

Supplement: Figure 4—figure supplement 1—source data 1. [file elife-103537-fig4-figsupp1-data1.zip › Figure 4 - supplement 1 - source data-01.png]

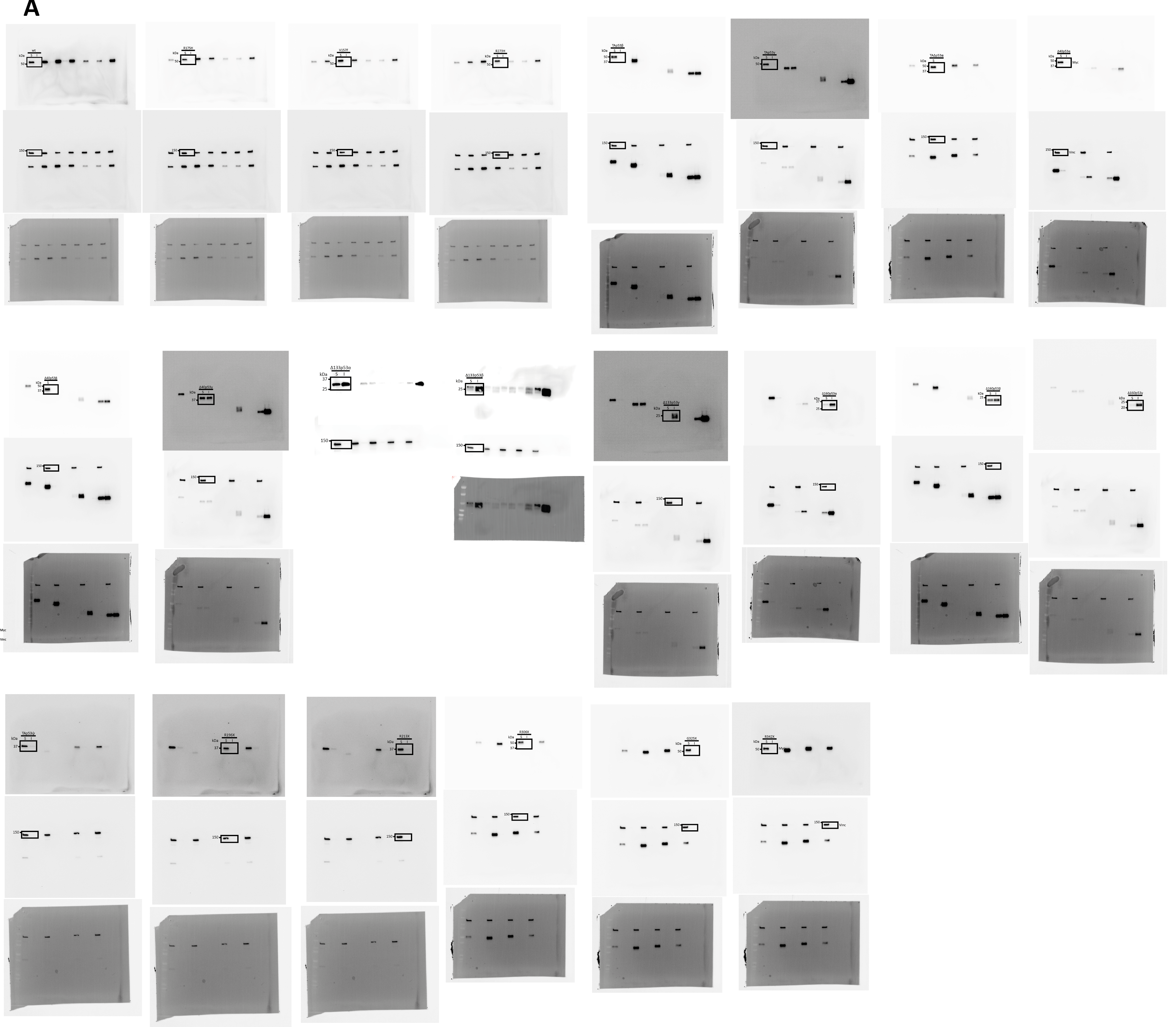

Supplement: Figure 4—figure supplement 2—source data 1. [file elife-103537-fig4-figsupp2-data1.zip › Figure 4 - supplement 2 - source data-01.png]

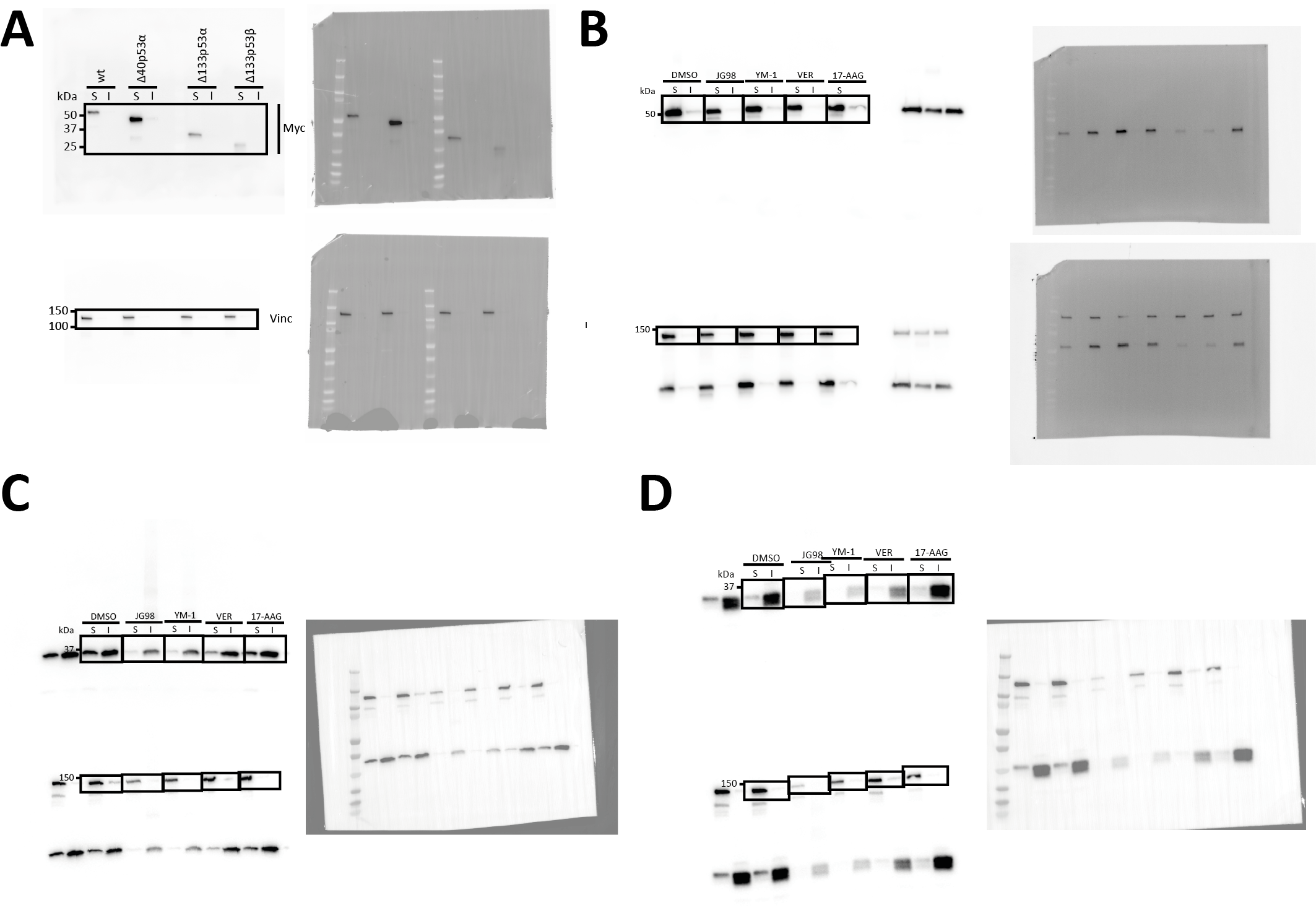

Supplement: Figure 4—figure supplement 3—source data 1. [file elife-103537-fig4-figsupp3-data1.zip › Figure 4 - supplement 3 - source data-01.png]

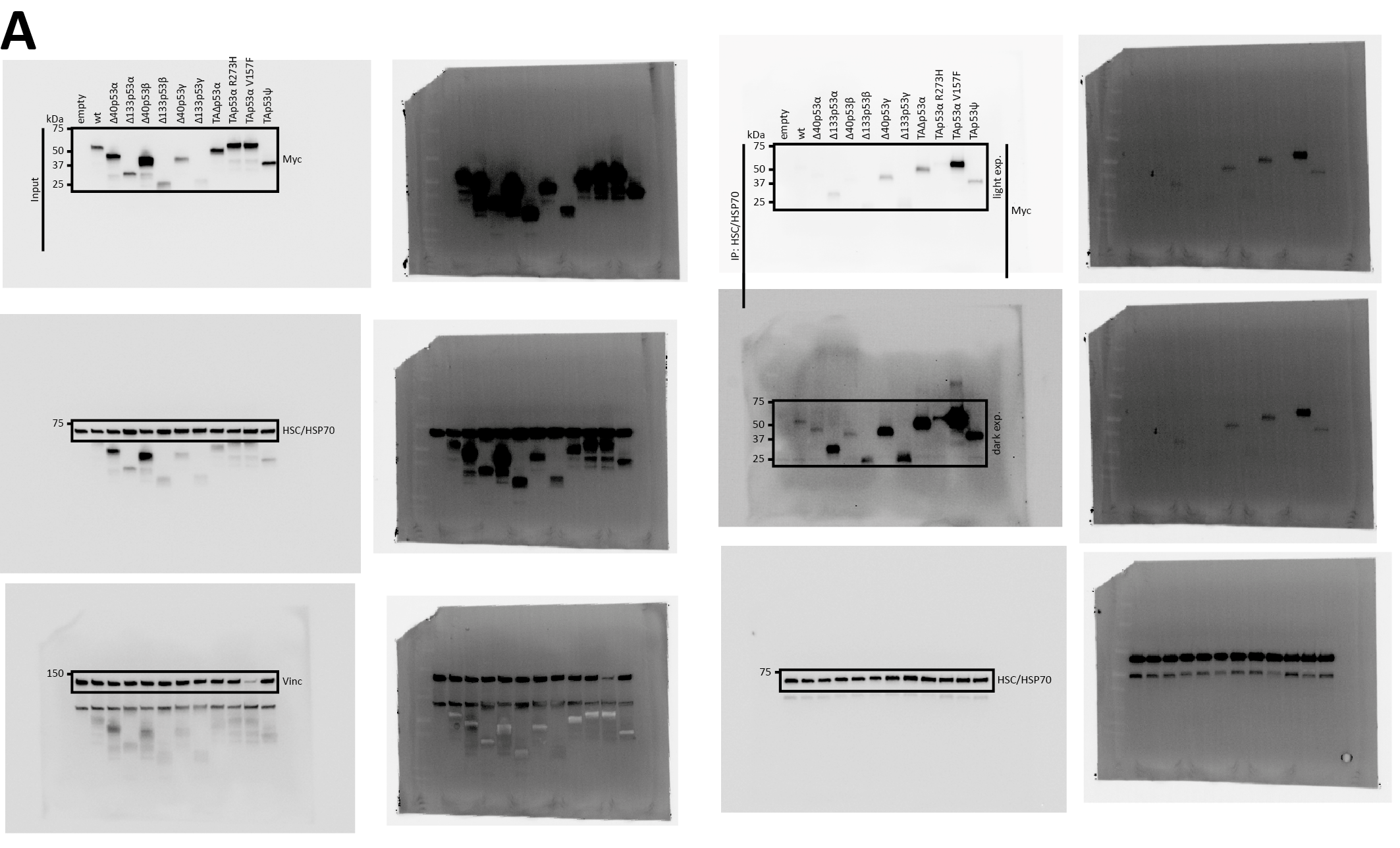

Supplement: Figure 5—source data 1. [file elife-103537-fig5-data1.zip › Figure 5 - source data-01.png]

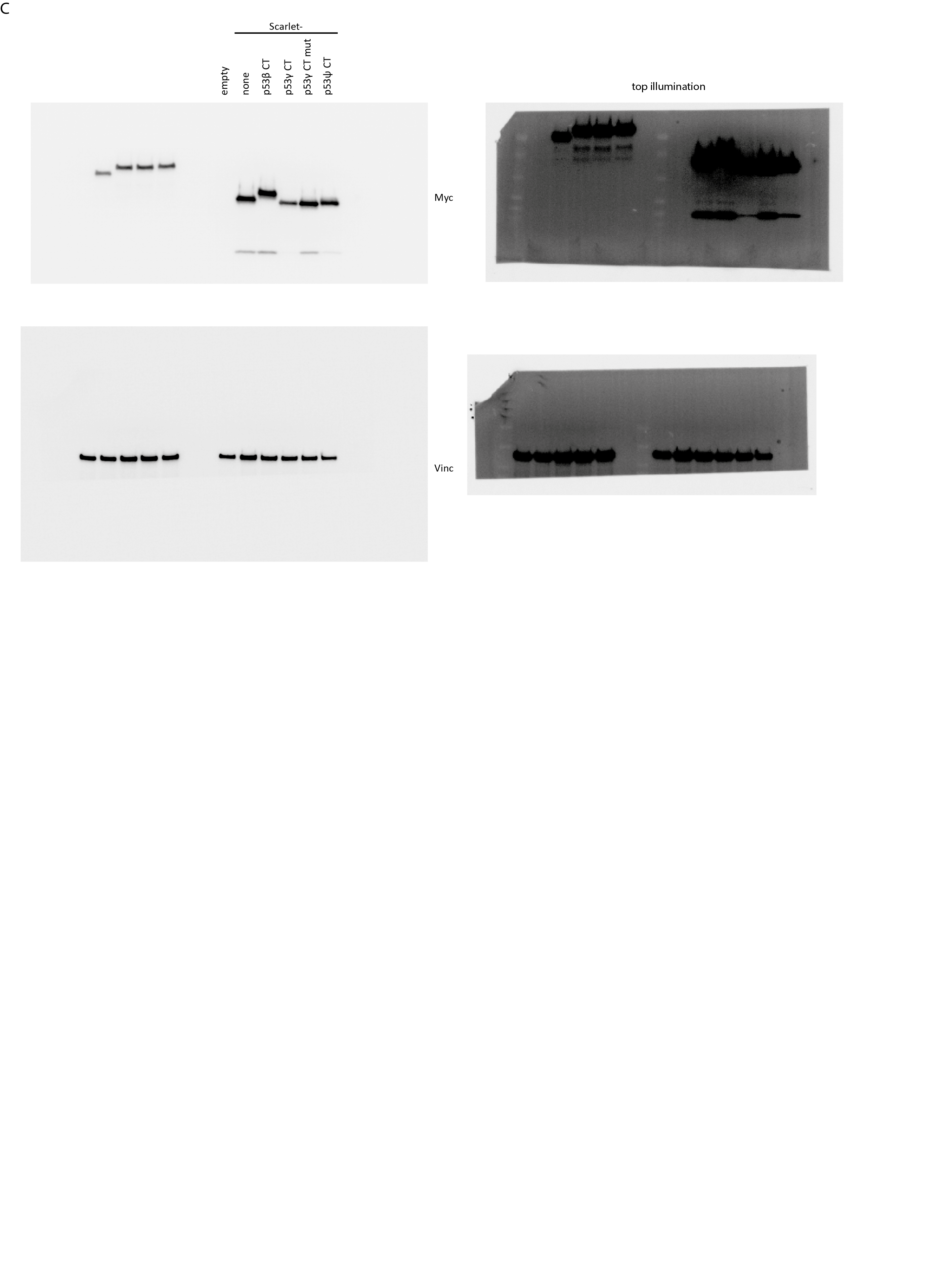

Supplement: Figure 5—figure supplement 1—source data 1. [file elife-103537-fig5-figsupp1-data1.zip › Figure 5 - supplement 1 - source data_C.png]

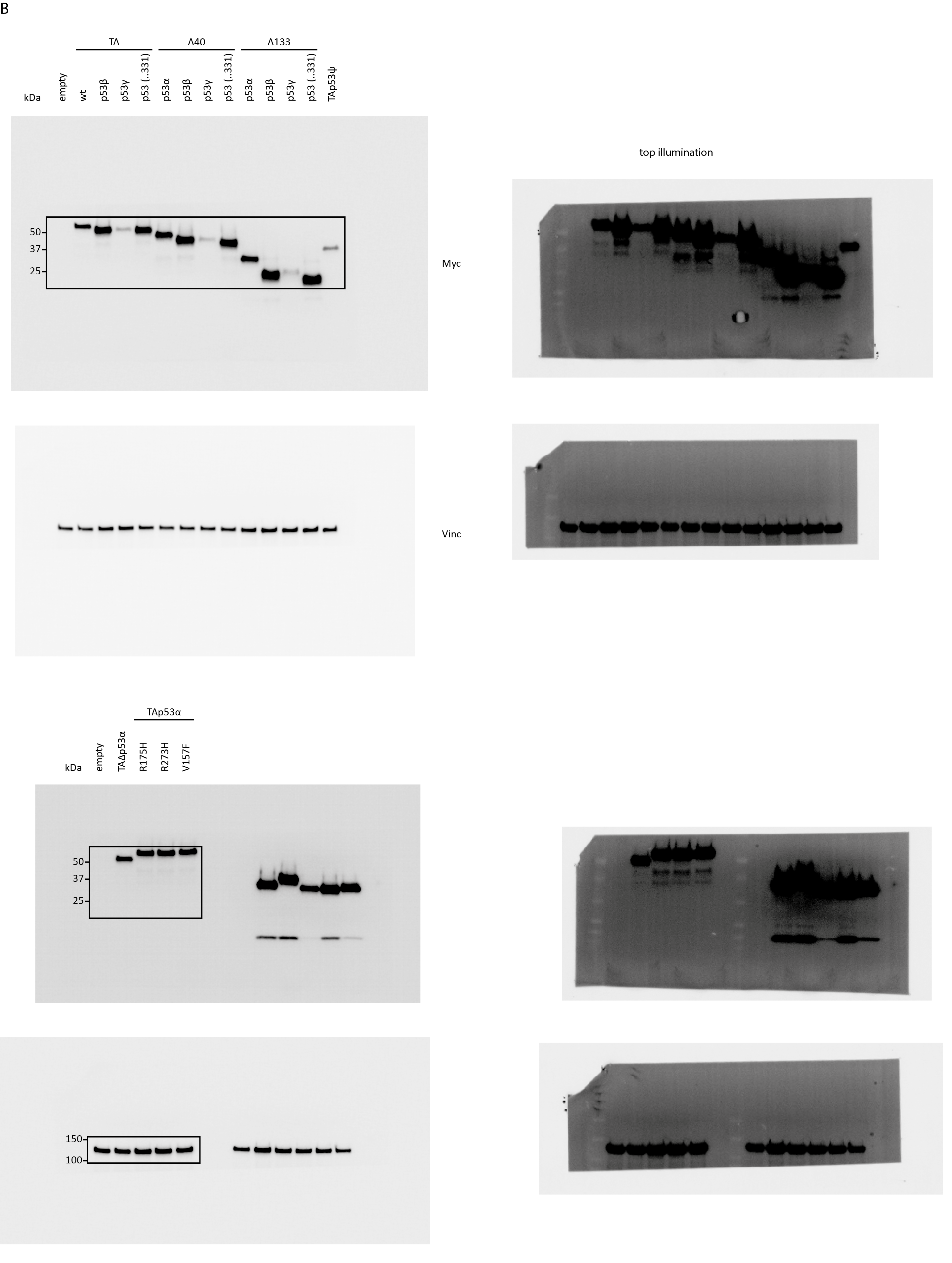

Supplement: Figure 5—figure supplement 1—source data 1. [file elife-103537-fig5-figsupp1-data1.zip › Figure 5 - supplement 1 - source data_B.png]

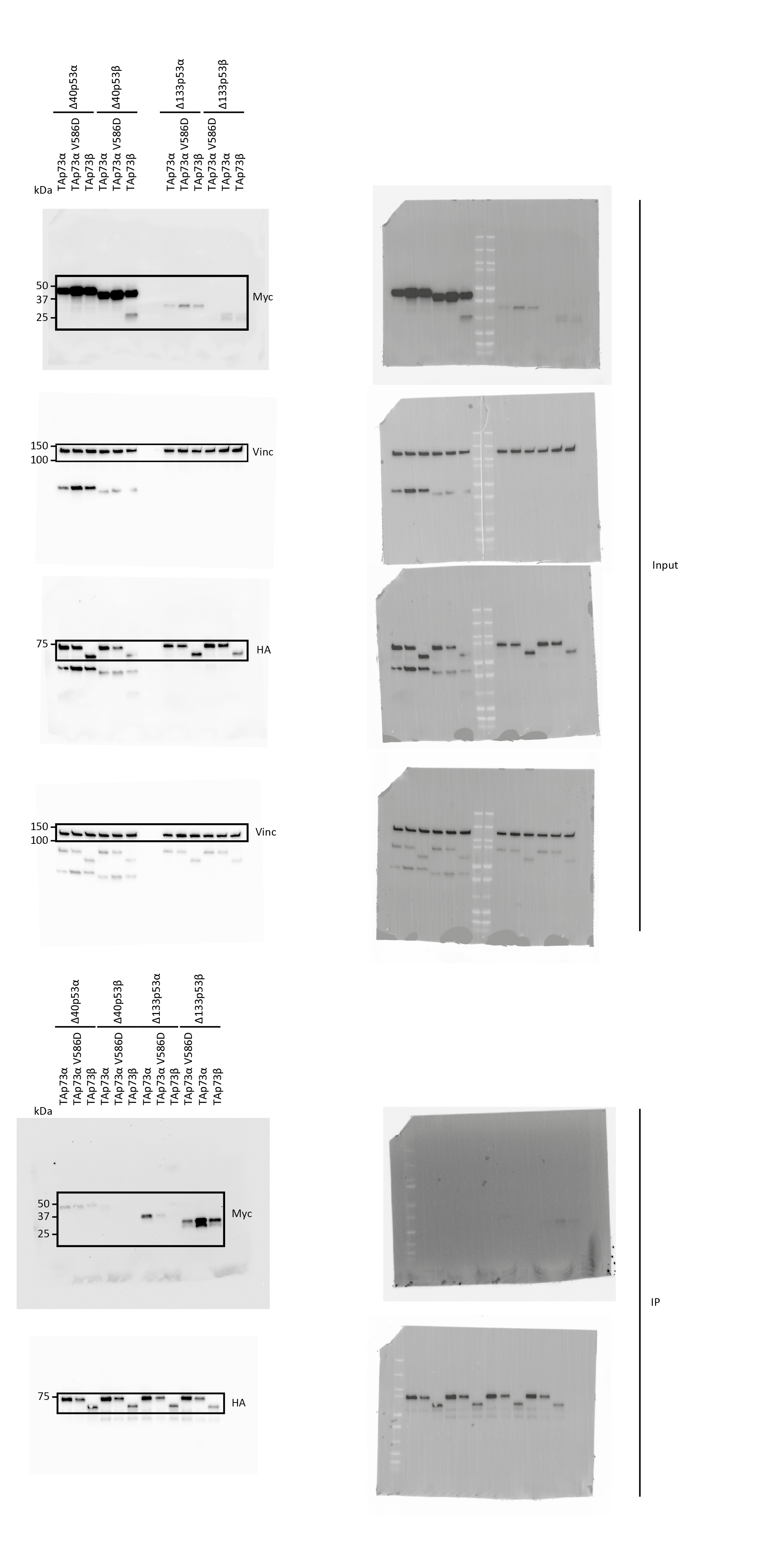

Supplement: Figure 6—source data 1. [file elife-103537-fig6-data1.zip › Figure 6 - source data_F-3.png]

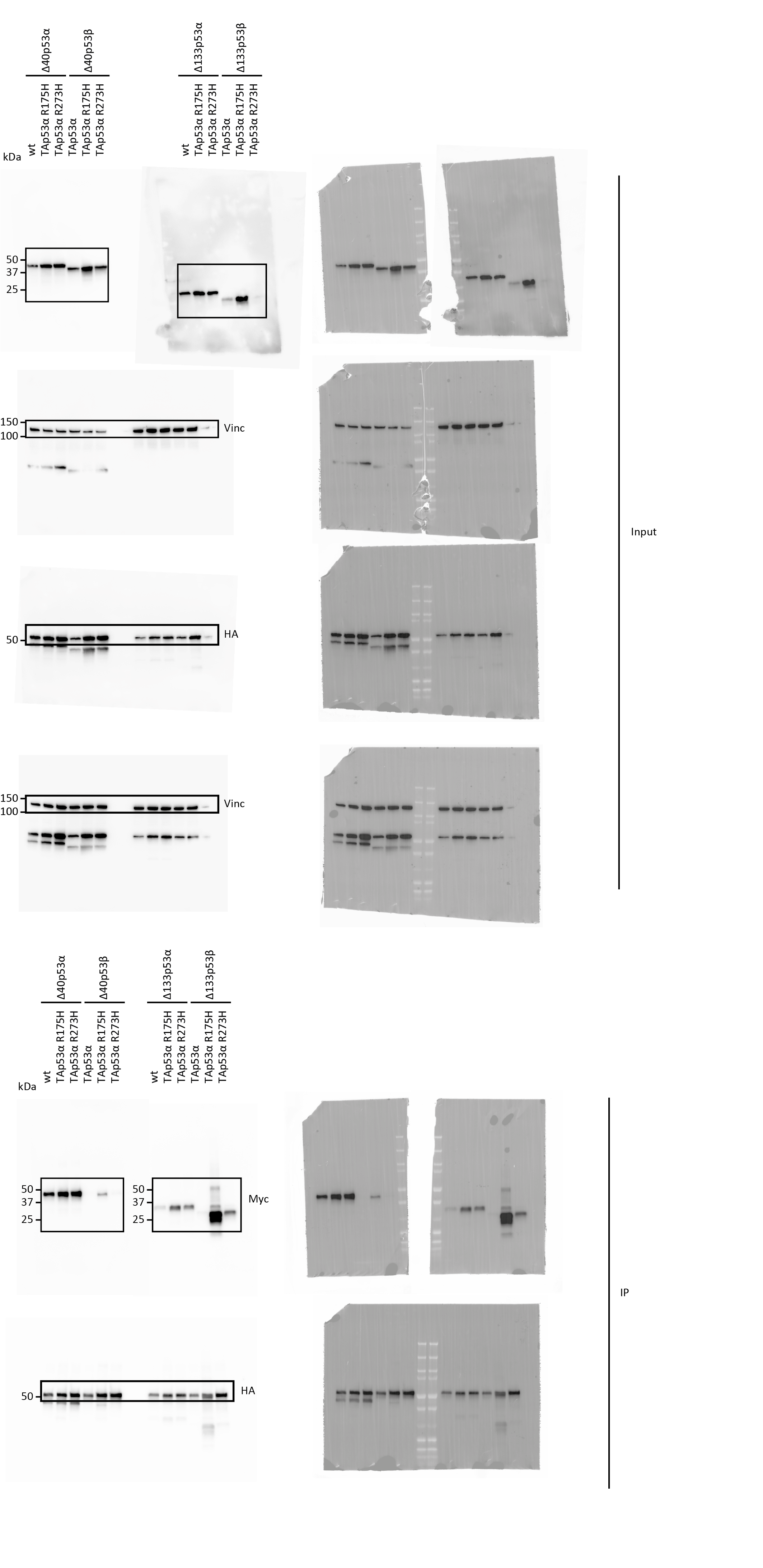

Supplement: Figure 6—source data 1. [file elife-103537-fig6-data1.zip › Figure 6 - source data_F-1.png]

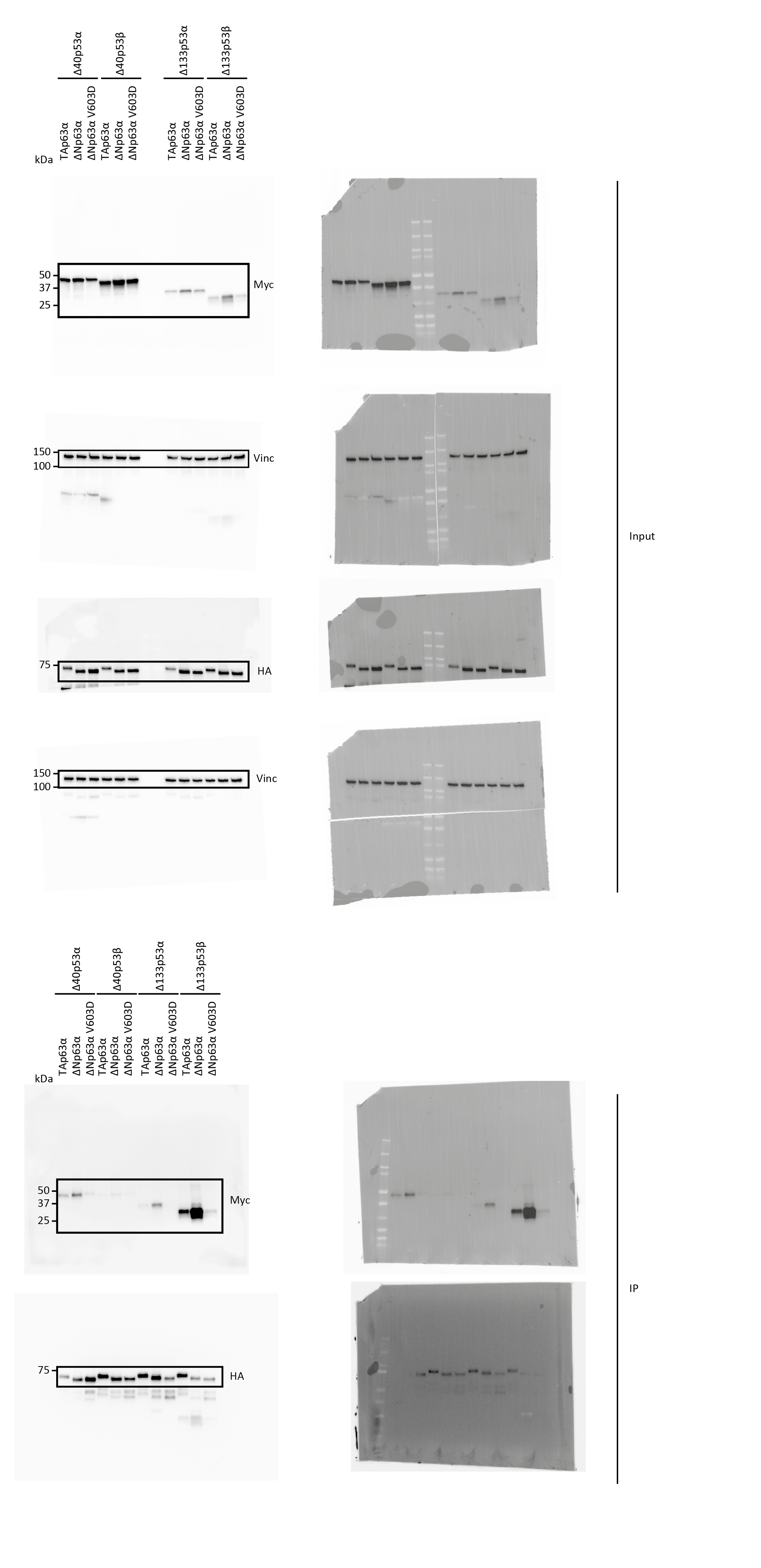

Supplement: Figure 6—source data 1. [file elife-103537-fig6-data1.zip › Figure 6 - source data_F-2.png]

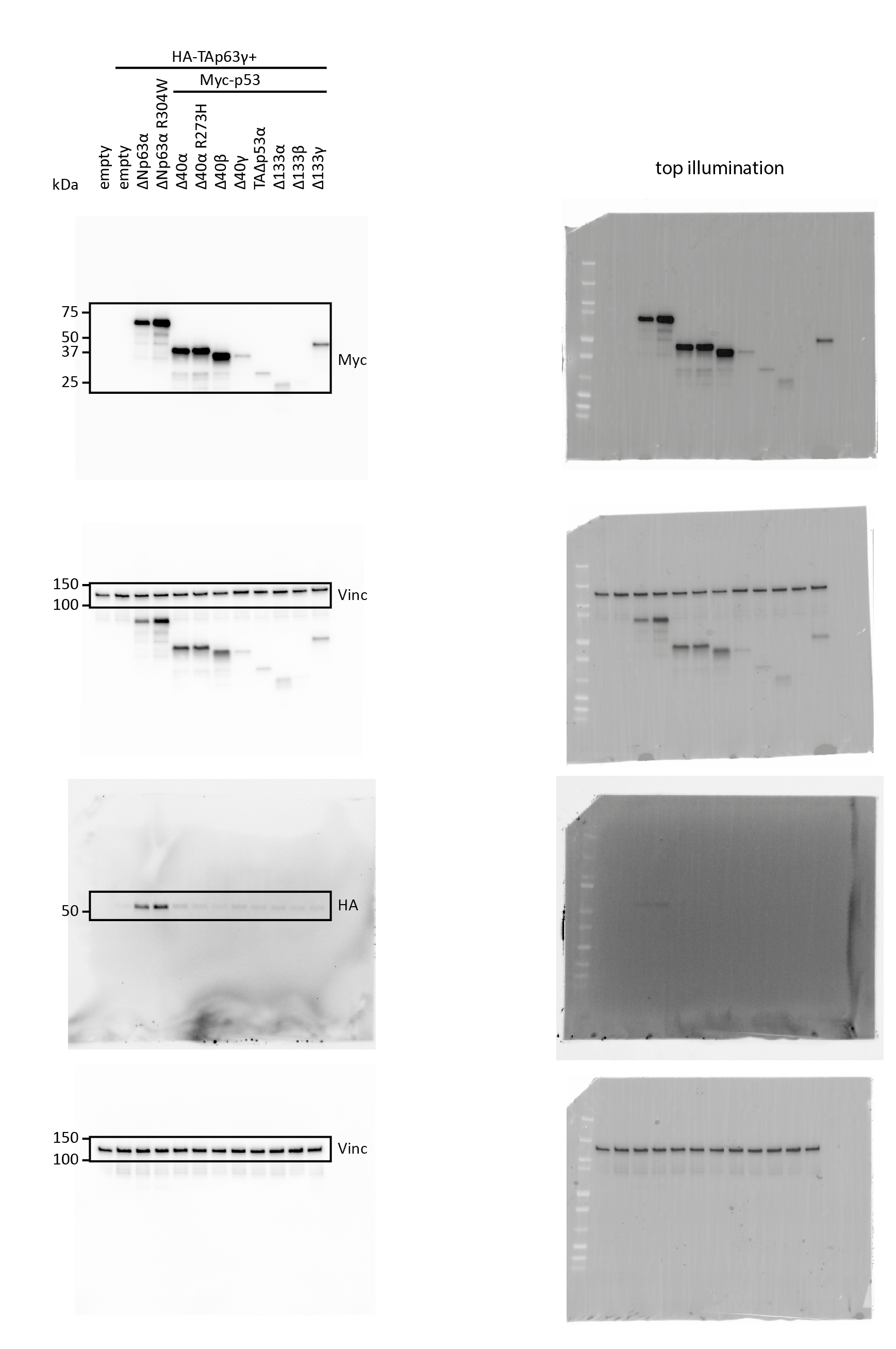

Supplement: Figure 6—figure supplement 1—source data 1. [file elife-103537-fig6-figsupp1-data1.zip › Figure 6 - supplement 1 - source data_D.png]

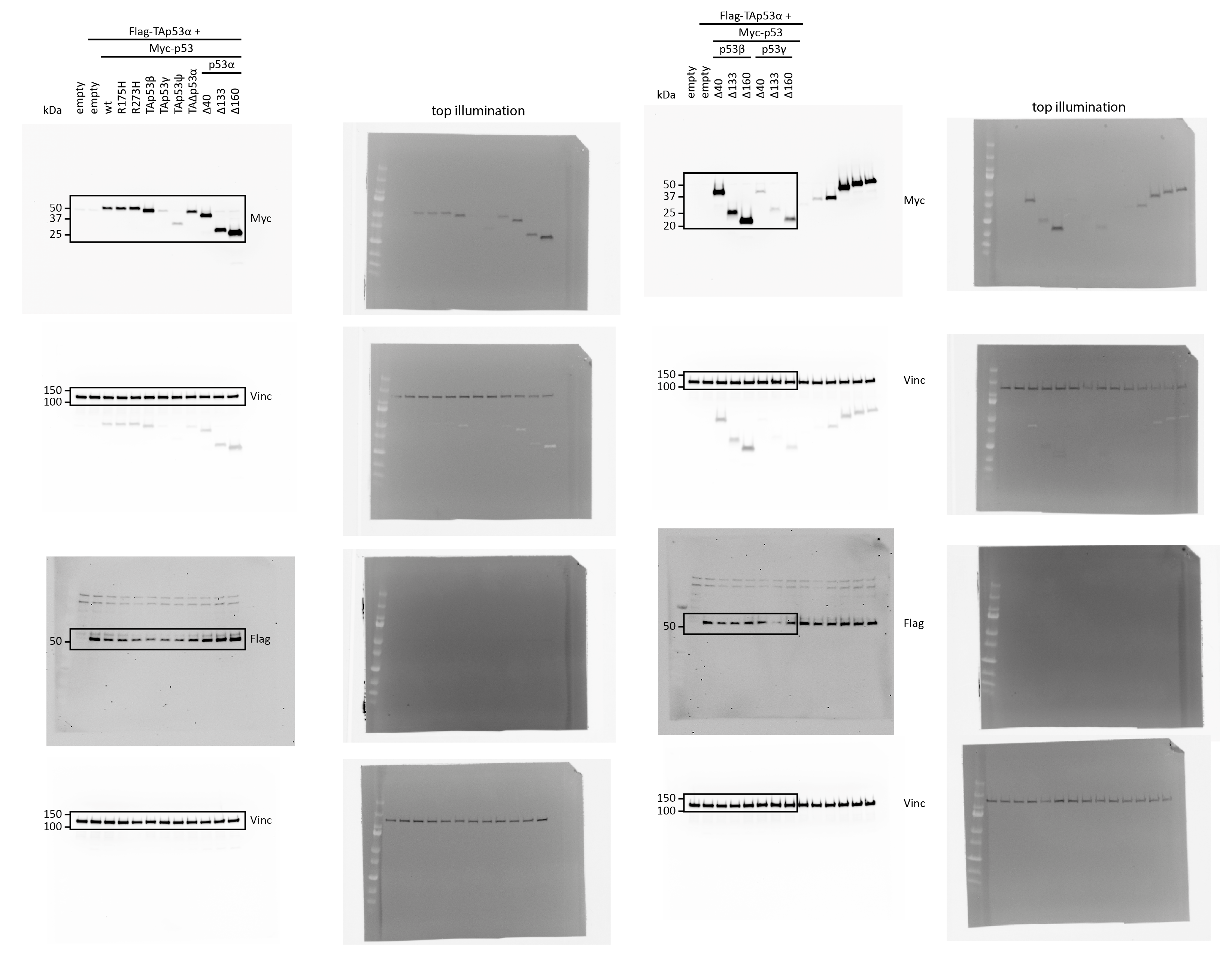

Supplement: Figure 6—figure supplement 1—source data 1. [file elife-103537-fig6-figsupp1-data1.zip › Figure 6 - supplement 1 - source data_A-1.png]

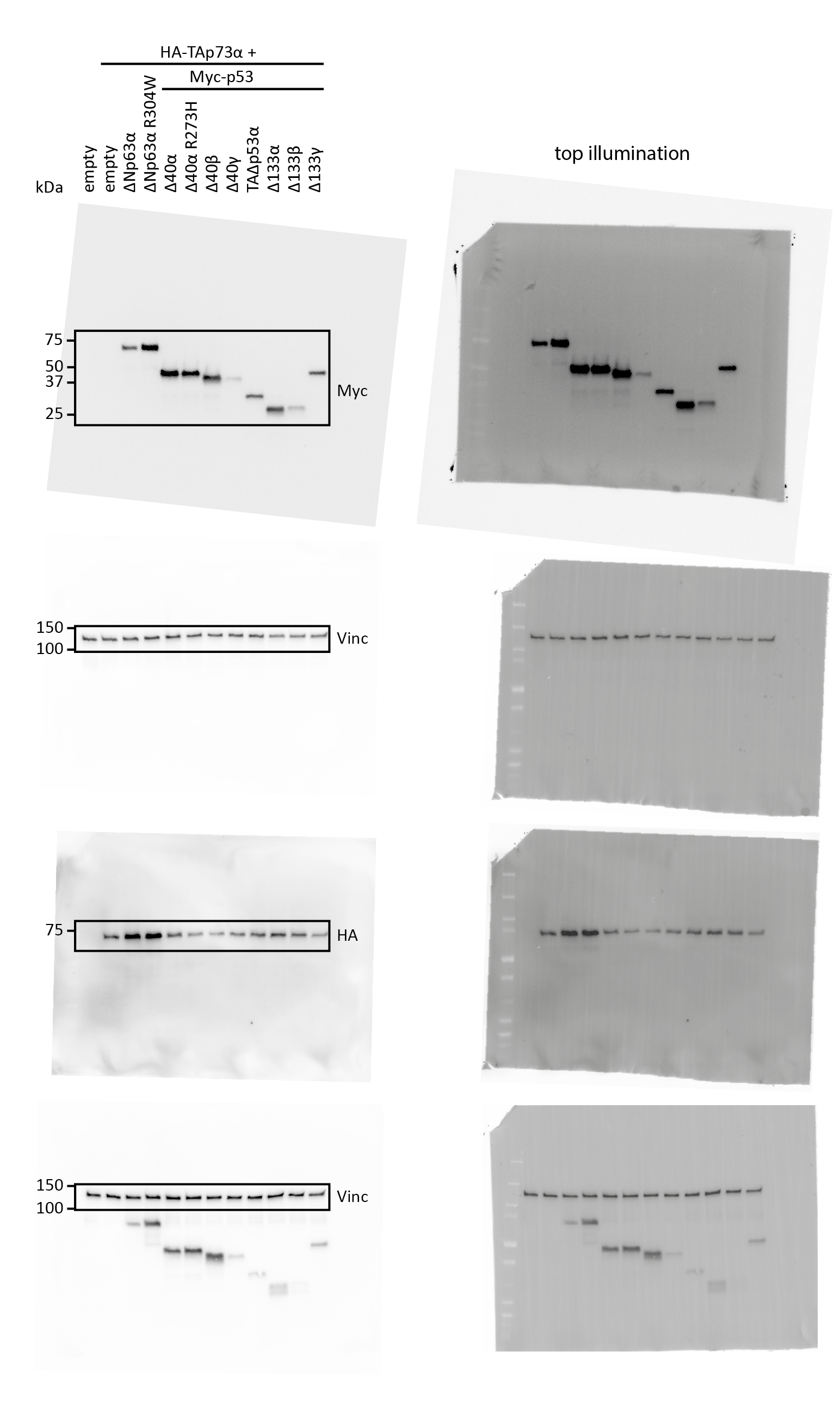

Supplement: Figure 6—figure supplement 1—source data 1. [file elife-103537-fig6-figsupp1-data1.zip › Figure 6 - supplement 1 - source data_B.png]

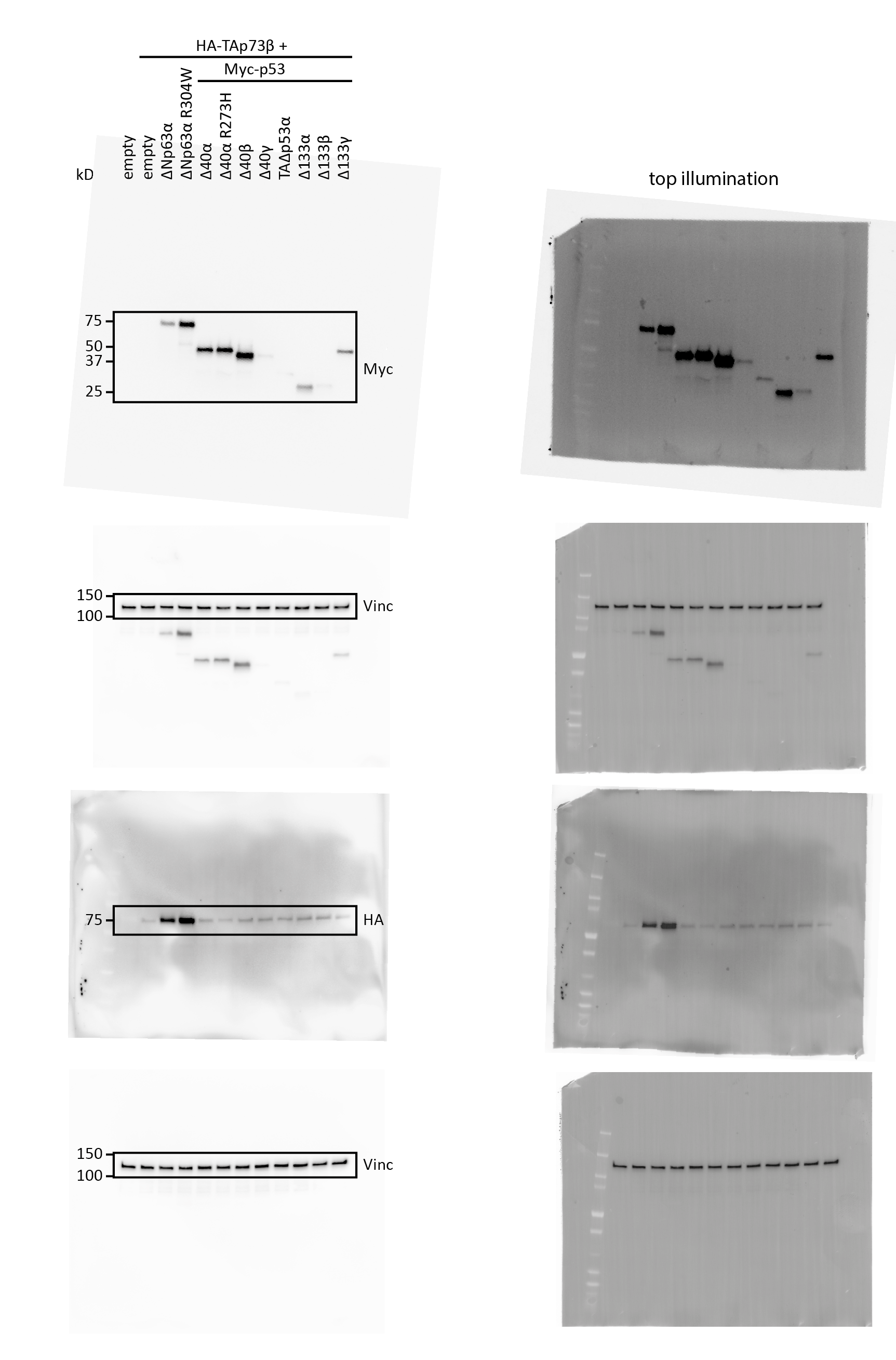

Supplement: Figure 6—figure supplement 1—source data 1. [file elife-103537-fig6-figsupp1-data1.zip › Figure 6 - supplement 1 - source data_C.png]
