# Supplementary material for: p53 isoforms have a high aggregation propensity, interact with chaperones and lack binding to p53 interaction partners
Source: eLife. 2025 Sep 10;13:RP103537. doi: 10.7554/eLife.103537 (PMC12422735; doi:10.7554/eLife.103537)
Supplement: Supplementary file 1. [file elife-103537-supp1.pdf]

Supplementary Table 1 Core protein hits for p53 isoforms associated with various chaperons *in-vivo*. Shown are the remaining core protein hits from Figure 5D and the enrichment for the respective p53 isoform. Displayed in bold are the only four hits all p53 isoform have in common (not shown in Figure 5D). Displayed are chaperons and other proteins associated with binding un-/misfolded proteins. Significant hits were determined by setting the parameters: log2 enrichment greater than or equal to 0.5 and p-value less than 0.05.

**Genes name**	**Enriched for**				
	Δ40p53α	TAp53β	TAp53γ	Δ133p53α	Δ133p53β
AHSA1	x			x	
CHAF1B	x	x		x	
CHORDC1	x	x	x	x	
CSNK2A1;CSNK2A3	x	x		x	x
DFFA	x	x	x	x	
DNAJA1	x			x	
DNAJA3		x		x	
DNAJB1	x		x	x	x
FKBP4	x	x	x	x	
HSPA1A;HSPA1B	x		x	x	x
HSPA4	x	x	x	x	
NPM1	x			x	x
NUDCD3	x		x	x	x
PDCD5	x	x		x	x
PPID	x		x	x	
PPIH	x	x		x	x
RUVBL2	x	x			x
**CHAF1A**	**x**	**x**	**x**	**x**	**x**
**NUDCD2**	**x**	**x**	**x**	**x**	**x**
**AIP**	**x**	**x**	**x**	**x**	**x**
**TP53**	**x**	**x**	**x**	**x**	**x**
